# The potential and limitations of large language models for automatic classification of teachers' motivational messages in educational research

**DOI:** 10.1111/bjep.70013

**Published:** 2025-08-03

**Authors:** Olivia Metzner, Yindong Wang, Gerard de Melo, Wendy Symes, Yizhen Huang, Rebecca Lazarides

**Affiliations:** ^1^ Department of Educational Sciences University of Potsdam Potsdam Germany; ^2^ Chair of Artificial Intelligence and Intelligent Systems Hasso‐Plattner‐Institute/University of Potsdam Potsdam Germany; ^3^ Institute for Psychology in Education, University of Münster Münster Germany; ^4^ Department of Research on Teaching and Teacher Education Kiel University Kiel Germany; ^5^ Science of Intelligence, Research Cluster of Excellence Berlin Germany

**Keywords:** artificial intelligence, classroom, large language models, motivational messages, natural language processing, teacher

## Abstract

**Introduction:**

The rapid advancement of artificial intelligence (AI) has created new opportunities in educational research, particularly in the efficient analysis of complex social interactions within classrooms. One promising area involves the classification of teachers' motivational messages. Traditionally, such assessments have relied on self‐reports and observer evaluations, which require a lot of staff and time resources. Recently, large language models (LLMs) have been employed to classify teachers' motivational messages, offering novel, less labour‐intensive approaches for classification.

**Aims:**

Building on these recent developments, this work presents a comprehensive literature overview exploring the applications, potential, and limitations of using LLMs to classify teachers' motivational messages.

**Results:**

The present comprehensive literature overview indicates that the use of LLMs for classifying teachers' motivational messages is a promising yet still emerging field of research. Recent studies have applied LLMs in innovative ways, drawing on established motivational theories and employing novel classification techniques, such as zero‐shot and few‐shot prompting or fine‐tuning, to classify motivational messages. Open questions remain, particularly concerning the structure, quantity, and quality of annotated material.

**Discussion:**

Whereas recent studies have demonstrated the potential of LLMs to offer scalable and time‐efficient alternatives for classifying motivational messages in the classroom, several challenges persist. These include concerns related to the quality and quantity of training data, model generalisability, the ability to capture the complexity of classroom interactions, and biases involved in integrating LLMs as a classification method. This comprehensive literature overview provides practical recommendations for the responsible use of LLMs in educational research and school practice.

## BACKGROUND

The rapid progress of artificial intelligence (AI) is furthering new developments in educational research. AI‐based methods enable researchers to assess and analyse large amounts of data in a time‐efficient manner and uncover underlying patterns in data on learning and interaction (Gardner et al., 2021; Wang et al., 2021). Due to its scalability and analytical power, AI is becoming an increasingly attractive tool for investigating educational processes that would otherwise be too time‐consuming or complex to analyse using traditional methods.

One domain in which the application of AI‐based methods in research is developing particularly rapidly is the assessment of social interaction processes in classrooms (Wang et al., 2022; Zhou, 2022). A central component of social interactions in classrooms is teachers' use of verbal messages in classroom discourse (Liu, 2021), such as their use of motivational messages (Putwain et al., 2021). Teachers' motivational messages can be defined as advisory messages that emphasise specific types of motivation aiming to encourage students to participate in school‐related activities (Metzner et al., 2025; Santana‐Monagas, Putwain, et al., 2022). Motivational messages from teachers present a particularly promising focus for AI‐based research, as their frequency, variability, and context‐dependency make them challenging to capture and analyse manually. Moreover, assessing social interactions in classrooms is highly complex because of their dyadic, bidirectional, dynamic, and often implicit character (Trauernicht et al., 2025; Wentzel, 2022).

To date, numerous studies assessing teachers' motivational messages as part of social interactions in class have relied on either teacher‐ or student‐reported data (Putwain et al., 2016; Santana‐Monagas, Putwain, et al., 2022) or observer‐reported data (Falcon et al., 2023; Reeve & Jang, 2006). Yet, each of these methods yields certain limitations, such as resource‐intensive data collection (Cash et al., 2012), social desirability effects (King & Bruner, 2000), and the influence of external factors, such as achievement, that may affect students' self‐reports (Göllner et al., 2018).

With the current developments in AI‐based text classification, novel alternative methods offer promising potential to complement traditional observer methods, enabling researchers to analyse large amounts of data on classroom discourse in a time‐efficient way. Such methods include, for example, traditional machine learning methods or deep learning methods based on neural networks (for an overview, see Tang et al., 2021). Large language models (LLMs) are deep learning models designed to interpret, generate, and process human language by predicting the probability of linguistic units (Gan et al., 2023; Wang, Chu, et al., 2024).

Current research has contributed valuable insights by incorporating LLMs' few‐shot ability to classify teachers' exam‐related motivational messages from students' open‐ended responses (Alqassab & León, 2024), applying zero‐shot prompting to assess teachers' encouragement and warmth in 16‐min transcript segments (Hou et al., 2024), and fine‐tuning pre‐trained LLMs to investigate teachers' gain‐ and loss‐framed messages (Falcon & León, 2024) or pre‐service teachers' SDT‐based motivational messages (Metzner et al., 2025) using teacher transcripts. So far, however, a comprehensive overview of the strengths, challenges, and integration of LLMs in research classifying teachers' motivational messages in classrooms is currently missing.

In the present work, we address this gap by providing a comprehensive literature overview on the applications, potential, and limitations of LLMs for classifying teachers' motivational messages. We begin by outlining traditional assessment methods, discussing their strengths and limitations in capturing teachers' motivational messages. Next, we introduce LLMs and review existing research on the use of LLMs in classifying such messages. Subsequently, we discuss the capabilities and constraints of LLMs and conclude by offering general recommendations for their application in classroom‐based message classification.

### Traditional assessment practices in the domain of teachers' motivational messages

In the following sections, we review three commonly used methods for assessing teachers' motivational messages, how these methods have been applied, the empirical findings that have emerged, and the strengths and challenges associated with employing such methods.

#### Student reports of teachers' motivational messages

A widely used approach to assess teachers' motivational messages in classrooms is the use of student self‐reports, typically collected through closed‐ended questions that allow students to evaluate the quality and frequency of teachers' messages (Putwain & Symes, 2011; Santana‐Monagas, Putwain, et al., 2022). Several instruments have been developed for this purpose, including the Teachers' Use of Fear Appeals Questionnaire (TUFAQ; Belcher et al., 2022; Putwain & Roberts, 2009; Putwain & Symes, 2011) and the Teachers' Engaging Messages (TEM) questionnaire (Santana‐Monagas, Núñez, et al., 2022; Santana‐Monagas, Putwain, et al., 2022). Research assessing teachers' motivational messages using closed‐ended student reports has shown, for example, that teachers' use of fear appeals (motivational messages that emphasise the consequences of failure) appraised by the students as threatening longitudinally increased students' worry and tension components of test anxiety, performance‐avoidance, and mastery‐approach goals (Putwain & Symes, 2011; for an overview, see Putwain et al., 2021). Student self‐reports are frequently used in educational research because of their strong predictive validity for students' learning outcomes, including their motivation and achievement (Wagner et al., 2016), and particularly closed‐ended questions enable the collection of data from large samples in a cost‐efficient manner (Brewer et al., 2015). However, closed‐ended questionnaires constrain respondents to predefined items, which may fail to fully capture the nuances of students' experiences and perspectives (Alqassab & León, 2024; Reja et al., 2003).

As an alternative to closed‐ended questions, researchers have employed open‐ended questionnaires, asking students to describe the messages their teachers use (Alqassab & León, 2024; Kaufmann et al., 2021). Studies using students' open‐ended reports to assess teachers' motivational messages have revealed that teachers' use of reassuring messages was positively and cross‐sectionally related to students' intrinsic motivation (Alqassab & León, 2024). Findings from open‐ended student reports are a valuable complement to closed‐ended formats, as they allow students to articulate their experiences in their own words and enhance the understanding of teachers' motivational message use without being directed by a predefined framework. However, open‐ended reports tend to exhibit higher non‐response rates and are more time intensive to code (Reja et al., 2003).

Although open‐ and closed‐ended student questionnaires are valuable tools for examining the relation between teachers' motivational messages and student outcomes, they also present several methodological challenges, in addition to those raised above. Responses to both types of questionnaires can be influenced by external factors unrelated to the actual teaching behaviour. First, students' personal feelings toward a teacher can be affected by how much they personally like the teacher being evaluated—a phenomenon known as leniency bias (Podsakoff et al., 2024). For instance, Clayson (2022) found that university students' evaluations of their instructors' teaching were affected by how much they liked each instructor. In the context of teachers' use of motivational messages, this could mean that students might fail to detect some or all types of teachers' motivational messages due to being distracted by their interpersonal relationship with the teachers.

Second, leveraging self‐report assessments for both predictors and outcomes of teachers' messages might lead to an overestimation of effects caused by common method biases (Podsakoff et al., 2003). For example, Santana‐Monagas, Putwain, et al. (2022) used closed‐ended questionnaires to assess both students' perceptions of their teachers' engaging messages and the students' motivation to learn. In such cases, relying on student reports for both the predictor (e.g., teachers' motivational messages) and outcome variables (e.g., students' learning motivation) may introduce bias, as responses can be influenced by factors such as response style—the tendency to agree or disagree with items regardless of their actual content (Podsakoff et al., 2024). To summarise, whereas student reports offer valuable insights on teachers' motivational messages use, they may be limited by external factors such as students' personal feelings towards teachers or prevalent response styles among participants.

#### Teacher reports of teachers' motivational messages

In addition to students' reports of teacher messages, teachers' self‐reports have been used to assess teachers' motivational messages (Putwain et al., 2016; Putwain & von der Embse, 2018; von der Embse et al., 2017). For instance, Putwain et al. (2016) adapted their previously developed TUFAQ student‐report instrument by rephrasing the items to allow teachers to self‐report the frequency of their fear appeals. In a study by von der Embse et al. (2017), the authors modified the instrument further to tailor its items specifically to standardised testing contexts. As a result of this adjustment, teachers were able to validate their own teaching practices based on both their pedagogical knowledge and personal judgements—elements not accessible to students (Fauth et al., 2014, 2020).

Teacher reports using closed‐ended questionnaires are a valuable method for assessing motivational messages, as they provide insight into how such messages relate to individual teacher characteristics. For example, Putwain and von der Embse (2018) have shown that teachers' self‐reported frequency of fear appeals in class was negatively related to their self‐efficacy for student engagement. Additionally, von der Embse et al. (2017) showed that teachers who experienced higher stress and perceived tests as less valuable were more likely to use fear appeals. These findings highlight that teachers' self‐reports not only reveal patterns in motivational message use but also relate meaningfully to motivational variables, such as their self‐efficacy beliefs, that influence their motivational message use.

Despite their usefulness, teacher self‐reports also have notable limitations, particularly due to various response biases that can affect the accuracy of the data. One common limitation is social desirability bias, the tendency of respondents to present themselves in a favourable light in alignment with social norms (King & Bruner, 2000). In the context of teachers' motivational message use, teachers may underreport, for example, their use of fear appeals, as such strategies may be perceived as less socially acceptable compared to gain‐framed messages that emphasise student achievement and potential (Putwain & Roberts, 2012).

An additional limitation could be that teachers may tend to attribute successes to internal factors and failures to external ones (McAllister, 1996). As a result, teachers may be more inclined to attribute students' learning success to internal factors, such as their own use of supportive messages. In contrast, they may attribute students' failures to external factors, rather than to potentially detrimental practices teachers themselves may have employed, such as the use of fear appeals.

Furthermore, in a qualitative study, Urdan (2004) found that teachers' self‐reported beliefs showed no clear correspondence with their motivational messages use. Thus, it might be difficult to accurately capture teachers' intentions regarding their motivational message use through questionnaires and to align these intentions with their actual observed messages in the classroom. In summary, even though teacher reports offer the opportunity to include teachers' own perspectives when evaluating their teaching practices, teachers' self‐reports can be distorted through their own biases.

#### Observer reports of teachers' motivational messages

In addition to student and teacher self‐reports, observer reports offer a valuable alternative for assessing teachers' motivational messages. Observer reports are considered an effective method for assessing teaching behaviours (Helmke, 2009) and involve trained raters who observe in‐person or recorded classroom lessons and evaluate teaching behaviours based on the material. For instance, Wilkinson et al. (2020) conducted an in‐person exploratory observational study of 30 lessons, collecting various types of teachers' messages, including reassurance messages and efficacy appeals. Similarly, Falcon et al. (2023) analysed classroom audio recordings, with human raters annotating transcripts based on the Teachers' Engaging Messages (TEM) framework, which categorises teachers' messages into gain‐ and loss‐framed messages as well as motivational appeals based on self‐determination theory (SDT; Deci & Ryan, 2008; Santana‐Monagas, Putwain, et al., 2022). Their study found that teachers with higher‐performing students were rated as using engaging messages more frequently and were particularly likely to use engaging messages with an extrinsic appeal. The studies conducted indicate that both in‐person and pre‐recorded classroom settings can serve as valuable sources for assessing teachers' motivational messages.

Observer reports have also been used to explore teachers' motivational messages, which promote students' innate need for autonomy, competence, and relatedness—theoretically grounded in SDT (Deci & Ryan, 2000, 2002). Reeve and Jang (2006), for instance, tested in an experimental study whether the use of autonomy‐supportive or ‐controlling instructional behaviour affected students' perceived autonomy. The 10‐min video‐recorded one‐to‐one sessions were conducted with pre‐service teachers randomly assigned to either the teacher or student role. The results showed, for example, that providing encouragement—in this study classified as an autonomy‐supportive behaviour—was positively linked to students' perceived autonomy. In contrast, teachers' controlling behaviours, such as controlling questions and “should/got to” statements, were negatively associated with students' perceived autonomy. Metzner et al. (2025) additionally utilised SDT and transcribed video recordings of pre‐service teachers, classifying their motivational messages as supporting or thwarting students' need for autonomy, competence, and relatedness (Ahmadi et al., 2023; Deci & Ryan, 2000). The authors found, for example, that pre‐service teachers' self‐efficacy for classroom management increased the frequency of relatedness‐supportive messages used. These studies demonstrate that observer reports, when guided by a theoretically grounded framework, such as SDT, provide a robust method for capturing teachers' motivational messages.

In addition, both studies not only employed SDT as a theoretical framework to classify motivational messages, but also used pre‐service teachers as their sample to be analysed. Investigating pre‐service teachers' messages is particularly valuable, as their limited teaching experience may result in the use of different types of motivational messages compared to in‐service teachers. For example, Holstein et al. (2022) found that pre‐service teachers provide feedback about the process less frequently than in‐service teachers—a motivational message type which can be assigned as a competence‐supportive message (Ahmadi et al., 2023; Metzner et al., 2025). Focusing on the use of motivational messages by pre‐service teachers offers valuable opportunities for teacher education programs to effectively support the implementation of supportive rather than undermining messages in their teaching practice.

Despite their strengths, observational methods face notable practical and methodological challenges that can limit their effectiveness in educational research. One major challenge is that observational methods are resource‐intensive, requiring substantial effort to collect classroom data and train human raters to classify the data reliably (Cash et al., 2012). Because of these demands, studies often include only a limited sample of lessons per teacher and may therefore fail to capture the full range of teaching behaviour used across diverse instructional scenarios (Praetorius et al., 2014), such as the variability of teachers' motivational messages used. This limited sampling increases the risk of overgeneralising teachers' behaviour based on a narrow set of observations (Clausen, 2002). These constraints suggest that, while observational methods can yield rich and detailed insights into teachers' use of motivational messages, their resource‐intensive nature may limit data representativeness, thereby potentially compromising the validity and generalisability of the findings.

### LLMs as tools for classifying teachers' motivational messages

Taken together, student and teacher self‐reports, along with observer reports, are valuable instruments for assessing teachers' motivational messages. However, each approach presents methodological challenges that must be taken into account. For instance, self‐reports may be affected by biases such as personal feelings toward the individual being evaluated (e.g., leniency bias; Podsakoff et al., 2024) or social desirability effects (King & Bruner, 2000). Observer reports, on the other hand, are often resource‐intensive (Cash et al., 2012) which can limit sample sizes and reduce scalability (Clausen, 2002). To overcome these limitations and advance current research practices, AI‐based systems—such as LLMs—can be employed to address the current challenges.

The following section provides an overview of the application of LLMs to classify teachers' motivational messages. The section begins with a brief introduction to the technical foundations of LLMs in comparison to traditional text classification models. Subsequently, it outlines how approaches such as fine‐tuning can be used to combine task‐specific data with the general capabilities of LLMs to classify teachers' motivational messages. Following this, four recent studies employing LLMs as a classification method are reviewed, each applying different methodological and motivation‐theory‐based approaches.

#### General technical foundations of LLMs

One potential complement to traditional observer‐reported classification of teachers' motivational messages is LLMs such as GPT‐4 from OpenAI (OpenAI, 2023), LLaMA from Meta (Touvron et al., 2023), and Gemini from Google (Anil et al., 2023).

A key advantage of LLMs over traditional text classification methods is their ability to model context‐dependent meaning through deep, contextualised representations of language data. Whereas conventional machine learning approaches often rely on fixed, context‐independent representations, LLMs use self‐attention mechanisms to compute context‐dependent representations of language (Demszky et al., 2023; Kamath et al., 2024). This mechanism allows the model to assign dynamic weights to different tokens in the input sequence based on their contextual relevance, enabling it to capture long‐range dependencies within text (Amaratunga, 2023). LLMs' context sensitivity could be particularly effective for capturing the nuances and variability of teacher–student discourse.

Another strength of the LLMs lies in the variety and volume of their training data. LLMs are trained on extensive corpora of text data, sourced from diverse text categories such as books, journal articles, and websites, but also social media. With improved modelling and an increased amount of diverse training data sources, LLMs are capable of interpreting, processing, and generating human‐like language (Demszky et al., 2023; Wang, Chu, et al., 2024; Wei et al., 2022). As a result, LLMs could be capable of interpreting various types of classroom discourse, such as teachers' motivational messages.

From a technical perspective, LLMs generally involve a transformer neural network architecture, consisting of input embeddings, multiple transformer layers, and task‐specific output components (Vaswani et al., 2017; Wang, Chu, et al., 2024). The input processing begins by splitting text sequences into linguistic units called tokens, which are then converted into numerical vectors that combine semantic meaning (word embeddings) and positional information (positional encoding). The core transformer layers use self‐attention mechanisms to model relations between different parts of the input sequence, allowing the model to dynamically weight the importance of each token based on its relevance to other tokens in the context. Finally, the output layer generates the most probable token based on the learned contextual patterns (Vaswani et al., 2017). This architecture enables LLMs to capture complex linguistic patterns and uncover deeper semantic relations, which could be crucial for understanding teachers' use of motivational messages.

LLMs are pre‐trained on large, diverse corpora to capture general language patterns. However, to enhance LLMs' performance on domain‐specific tasks, fine‐tuning LLMs can notably improve their effectiveness (Brown et al., 2020; Kamath et al., 2024). During the fine‐tuning process, a domain‐specific labelled data set is used to adapt the model to the specific task (Kamath et al., 2024). In the context of classifying teachers' motivational messages, fine‐tuning a pre‐trained LLM with a labelled data set of teachers' messages could enable the development of a resource‐efficient tool for automatic classification. This approach potentially leads to the development of automated tools that classify teachers' motivational messages with minimal human effort, increasing efficiency while maintaining reliability in analysis.

Despite these advantages, fine‐tuning LLMs presents several practical challenges. For instance, running and fine‐tuning a pre‐trained LLM is a computationally intensive process, requiring access to high‐performance hardware and high‐quality annotated data to enable effective adaptation (Demszky et al., 2023). In the case of teacher motivational messages, limited computational resources or insufficient labelled data may reduce the effectiveness of fine‐tuning.

However, whereas LLMs require greater computational resources than traditional text classification models during both training and inference, they are more effective at processing linguistically complex data due to their model architecture and exposure to large‐scale pretraining corpora (Cunha et al., 2025; Demszky et al., 2023). Therefore, they may be more suitable for reducing the need for manual annotation effort, particularly in tasks such as analysing authentic classroom conversations.

#### Empirical evidence on LLMs as tools for classifying teachers' motivational messages

Integrating LLM‐based tools into research practices has gained interdisciplinary interest (Ding et al., 2024), including educational research (Yan et al., 2024). Recently, studies have applied LLMs to classify teachers' motivational messages, employing strategies that include zero‐ or few‐shot prompting and fine‐tuning of pre‐trained models to identify various types of teachers' motivational messages. The following two sections provide an overview of four studies, outlining their methodological approaches and empirical findings.

##### Zero‐shot and few‐shot prompting approaches to classify teachers' motivational messages

Zero‐shot and few‐shot prompting utilises pre‐trained LLMs to perform specific tasks without modifying the models' parameters. In these approaches, LLMs are guided using natural language prompts that specify the desired task. The key distinction between zero‐shot and few‐shot prompting is based on the number of examples included in the prompt. Zero‐shot prompting operates without any example annotations, relying solely on task instructions provided in the prompt, whereas few‐shot prompting combines the instructional prompt with a small set of carefully selected examples to guide the model's response (Brown et al., 2020; Kamath et al., 2024).

A key study involving a few‐shot prompting approach is one by Alqassab and León (2024). The authors used ChatGPT (GPT‐4) to classify students' open‐ended responses regarding their teachers' exam‐related motivational messages (e.g., fear appeals, reassuring messages). In their study, the authors developed a prompt which included coding instructions along with a few examples per category, allowing the model to infer the labelling criteria from contextual patterns. ChatGPT's ratings in comparison to human ratings achieved an overall inter‐rater reliability (Krippendorff's *α*) of .80. Key findings were that the majority (89%) of the messages used by teachers before exams were effort, capability, or reassuring messages, as opposed to fear appeals. Reassuring messages were positively and statistically significantly associated with students' intrinsic motivation. Their results showed that few‐shot prompting with ChatGPT can classify teachers' exam‐related motivational messages reliably and efficiently, with low computational cost. However, the authors relied on students' open‐ended reports rather than authentic classroom data, which may not accurately represent teachers' actual use of motivational messages. Additionally, the use of a proprietary model like ChatGPT limits transparency and reproducibility, making it difficult to validate or replicate the findings independently.

Building on a similar approach, Hou et al. (2024) used data from the international TALIS study where lesson videos were double‐rated on a 4‐point scoring scale in terms of the quality of teaching using 16‐min segments (OECD, 2020). The authors employed ChatGPT (GPT‐3.5 and GPT‐4) to rate the 16‐min segments based on teacher encouragement and warmth with pre‐developed prompts in a zero‐shot manner, i.e., without any fine‐tuning. The GPT‐4 ratings positively and moderately correlated with the human ratings (*r* = .34). The results demonstrate that LLMs can be reliably used as raters to assess teachers' motivational messages in terms of encouragement and warmth using authentic classroom data while at the same time requiring comparably low computational effort, similar to the approach Alqassab and León (2024) used. The authors' study makes a valuable and innovative contribution to the field by demonstrating the potential of LLMs in analysing teachers' motivational messages from teachers' transcripts. To further build on this important work, future research could consider finer‐grained segmentation, as nuanced teachers' messages can be diminished or lost within longer segmentation units.

##### Fine‐tuning a pre‐trained LLM for teachers' motivational messages classification

In contrast to zero‐ and few‐shot prompting, fine‐tuning adapts a pre‐trained LLM by updating its parameters using domain‐specific data. This enables the model to better adapt to the linguistic characteristics of teacher discourse by capturing its nuances and complexities (Demszky et al., 2023).

Highlighting the potential of fine‐tuning, Falcon and León (2024) fine‐tuned the Ada version of GPT‐3 and developed a two‐stage model for identifying and classifying teachers' engaging messages in classroom transcripts. In the first stage, the identification model differentiated between engaging messages and non‐engaging messages, achieving an F1‐score of .73 (recall 84.31%, specificity 97.69%, precision 64.40%). Second, the classification model classified the engaging messages as either gain‐ or loss‐framed messages, achieving an F1‐score of .89 (recall 91.11%, specificity 86.36%, precision 87.20%). Precision is the proportion of true positives among all predicted positives. Recall is the proportion of true positives among all actual positives. Specificity measures the proportion of correctly identified negatives among all actual negatives. The F1‐score, calculated as the harmonic mean of precision and recall, provides a balanced measure of a model's classification performance (Chinchor, 1992). The authors found that teachers used gain‐framed messages most of the time (67.33%) and employed loss‐framed messages less often (32.67%). Teachers' use of gain‐ and loss‐framed messages declined over the course of the school year. The authors' approach offers a meaningful contribution to the field, as they are among the few who have successfully adapted a pre‐trained LLM to classify teachers' motivational messages using authentic classroom data. Nevertheless, the authors employed a relatively broad classification system (e.g., gain‐ vs. loss‐framed messages) rather than classifying messages into more specific types of teachers' motivational messages. Building on this important work, a more fine‐grained and nuanced classification approach could further enhance LLMs' ability to capture a broader spectrum of motivational messages used by teachers.

Similar to Falcon and León (2024), Metzner et al. (2025) fine‐tuned a pre‐trained LLM to classify pre‐service teachers' SDT‐based motivational messages. The study used transcribed classroom data from 137 pre‐service teachers across 16 subjects, aiming to capture a broad range of motivational messages. Two trained raters annotated the transcripts using an SDT‐based motivational message coding scheme. The coding scheme was adapted from Ahmadi et al.'s (2023) classification system of teachers' motivational behaviours and reduced the initial 57 behavioural sub‐categories to 23 motivational message sub‐categories, organised under six main SDT‐based categories, namely autonomy‐supportive, competence‐supportive, relatedness‐supportive, autonomy‐thwarting, competence‐thwarting, and relatedness‐thwarting messages. Inter‐rater reliability for the overall coding scheme was *κ* = .73 (Brennan–Prediger), with category‐level *κ* values ranging from .61 to .78. The complete coding scheme and detailed inter‐rater reliability statistics for each category are presented in Metzner et al. (2025). In total, 2983 motivational message instances were identified, with over 80% classified as supportive and less than 20% as thwarting. The authors fine‐tuned the google/gemma‐2‐27b‐it model (Gemma Team, 2024) and used data augmentation to address imbalanced message distribution, generating additional thwarting messages via a zero‐shot prompting approach with ChatGPT (GPT‐4o). AI‐generated messages were reviewed by a blind rater and validated by two experts for authenticity. Fine‐tuning was performed using five independent train‐test splits and parameter‐efficient fine‐tuning (PEFT) with a LoRA adapter (Hu et al., 2021) to reduce computational costs. Further details on the fine‐tuning process can be found in Metzner et al. (2025). The fine‐tuned model achieved a robust performance when classifying supportive messages, with F1‐scores above .7 (autonomy‐supportive F1‐score .73 ± .01, competence‐supportive F1‐score .80 ± .03, relatedness‐supportive F1‐score .79 ± .04). In contrast, performance on thwarting message categories was lower, with F1‐scores below .7 (autonomy‐thwarting messages F1‐score .57 ± .06, competence‐thwarting messages F1‐score .27 ± .16, relatedness‐thwarting messages F1‐score .61 ± .05), indicating limited classification reliability for these categories. Additionally, the authors examined relations between LLM predictions of pre‐service teachers' supportive messages and their self‐efficacy beliefs. Pre‐service teachers with high self‐efficacy for instructional strategies expressed fewer relatedness‐supportive messages, whereas those with high self‐efficacy for student engagement expressed them more frequently. This study is among the first to fine‐tune an LLM using authentic classroom data from pre‐service teachers. Whereas the integration of SDT provides a strong theoretical foundation, the aggregation of 23 sub‐categories into six broad categories, combined with the highly imbalanced data distribution among the samples per category used for fine‐tuning, may risk oversimplifying diverse message types.

## DISCUSSION

This comprehensive literature overview explores the application, potential, and limitations of LLMs in classifying teachers' motivational messages. Recent studies have adopted innovative methods to classify these messages, drawing on diverse theoretical frameworks such as teachers' exam‐related motivational messages, teachers' encouragement and warmth messages, teachers' engaging messages, and pre‐service teachers' SDT‐based motivational messages. These studies have employed novel LLM‐based techniques, including zero‐shot and few‐shot prompting approaches, as well as fine‐tuning pre‐trained LLMs with authentic classroom data. The following section will further discuss the potential and limitations identified in current research and outline strategies to address the associated challenges facing future studies classifying teachers' motivational messages when applying LLM.

### Handling imbalanced and insufficient data when fine‐tuning an LLM

Our comprehensive literature overview outlines several main challenges when classifying teachers' motivational messages with LLMs. One key challenge is dealing with imbalanced data distributions and insufficient data in the training set. Imbalanced data distributions occur when the sample sizes of different categories vary notably, which can lead to lower model performance for minority categories (Ando & Huang, 2017; Buda et al., 2018). To address this issue, researchers often apply data balancing techniques, such as resampling (increasing the number of underrepresented categories) or reweighting (giving greater importance to minority categories during model training), to reduce bias towards majority categories (Kamath et al., 2024). Another novel approach involves data augmentation, a technique in which new data are generated—for example, by using AI to produce synthetic data (ValizadehAslani et al., 2024). However, AI‐generated data may not fully reflect authentic classroom discourse, as human conversations are often characterised by pauses, shifts in thought, and irregular sentence structures. As a result, AI‐generated messages may struggle to reflect the linguistic diversity and natural tone of authentic classroom discourse. Future studies could address this by using instructional prompts that not only guide LLMs to produce more human‐like language but also include a limited number of real examples of teachers' motivational messages, enabling a pre‐trained LLM to generate more realistic outputs through a few‐shot prompting approach.

Furthermore, studies may struggle to determine how many transcripts are needed to obtain a balanced number of motivational messages for LLM fine‐tuning and evaluation. To address this, future research could pilot the coding scheme on a smaller transcript subset to estimate the frequency of each message type. This preliminary analysis would help determine the sample size of transcripts needed to ensure a balanced representation of messages across all categories in the training data.

In the previous study, Metzner et al. (2025) addressed the problem of insufficient and imbalanced data for thwarting message categories by prompting ChatGPT in a zero‐shot setting to generate additional examples. However, this approach did not improve the model's performance, likely due to the AI‐generated messages lacking authenticity. Therefore, experimenting with few‐shot prompting to generate additional messages by incorporating instructional prompts with real examples of teachers' motivational messages may lead to more authentic data. Additionally, including prompt instructions that explicitly request teacher‐like messages using more natural language patterns might improve the quality of generated messages.

Taken together, effectively classifying teachers' motivational messages requires a balanced training and testing data set with a sufficient number of messages in each category. When data for minority categories are limited, strategies such as augmenting with AI‐generated messages can be employed; however, carefully designed prompts are essential to ensure the authenticity of the generated content.

### Challenges in human‐annotated data

Another key challenge is the quality of human‐annotated data for model training and testing. Several factors can influence data quality, including inconsistencies in labelled data, which may introduce noise and subsequently degrade model performance. Whereas deep learning models excel at recognising data patterns, excessive data variability may reduce accuracy when identifying linguistic characteristics within one category (Munappy et al., 2022). In the context of classifying teachers' motivational messages, a high variability of sub‐themes and sub‐patterns within a single category, combined with vaguely defined category definitions, may hinder model fine‐tuning and testing, resulting in reduced performance.

For example, Falcon and León (2024) fine‐tuned a classification model using teachers' gain‐ and loss‐framed messages and included messages that aimed to engage students in school tasks. However, this rather broad inclusion criterion may have introduced within‐category heterogeneity due to its coarse‐grained definition of engagement. To improve the model's interpretive accuracy, a more nuanced categorisation of engaging messages—such as the classification into various SDT‐based appeals alongside gain‐ and loss‐framed messages, as proposed by Falcon et al. (2023)—could enable more refined message interpretation.

Another example is the study by Metzner et al. (2025), which initially used 23 sub‐categories for human annotation, later merging them into six main categories for fine‐tuning. This consolidation may have introduced broad message variation within one category, contributing to reduced model performance. Moreover, unclear overlaps in sub‐category definitions likely affected both the quality of AI‐generated thwarting messages and the effectiveness of the fine‐tuning.

Taken together, achieving high model performance requires clearly defined and distinguishable categories, along with consistent and unambiguously assignable examples for each category. A lack of clarity and overly broad categories likely contribute to ambiguity in message classification and reduce reliability during model training. Whereas simplification is recommended, we still advocate for a classification framework grounded in theoretical foundations and empirical evidence to ensure validity.

Additionally, researchers could incorporate human‐in‐the‐loop (HITL) approaches during model development. In this approach, human practitioners iteratively review model outputs and provide corrections or adjustments before continuing training (Kamath et al., 2024). Employing HITL enables the model to assist in the annotation process while ensuring that human raters remain involved in key decision‐making steps.

### Generalisation of the results

Another important challenge is the limited generalisability of model performance across different classroom settings. The process of designing, collecting, and annotating data for model training takes place within a specific classroom context, where factors such as the school subjects, teacher background, and student composition may influence the resulting data (Wang, Tao, & Chen, 2024). For instance, data collected exclusively from mathematics lessons taught by highly experienced primary school teachers are likely to differ from data gathered across multiple subjects by novice teachers in a secondary school. In such cases, the classroom context in which the data are collected may influence the types of messages used by teachers, which in turn affects the initial structure of the coding scheme, shapes category definitions, and ultimately determines the composition of the training data set.

To ensure broader model applicability, it is important to evaluate the model's performance across varied educational contexts (Yan et al., 2024). For example, of the four studies reviewed in this work, two were conducted in Spanish schools (Alqassab & León, 2024; Falcon & León, 2024) and two in German schools (Hou et al., 2024; Metzner et al., 2025). Since the studies evaluated model performance using classroom data from different countries, linguistic and cultural factors may have influenced the types and frequency of motivational messages used.

In summary, classroom context can shape the structure and content of teachers' motivational messages, category definitions, and training and testing data, potentially limiting the model's generalisability. To ensure broader applicability, model performance should be evaluated across diverse educational and cultural settings.

### Complexity of classroom interactions

Furthermore, the complexity of classroom interactions, which is often shaped by context‐dependent human discourse, presents another key challenge. Mercer (2008) posits that classroom interactions are shaped by historical and dynamic aspects. The historical aspect encompasses institutional, cultural, and relationship influences among speakers, whereas the dynamic aspect refers to the spontaneous and evolving nature of discourse. These factors contribute to the complexity of classroom interactions—including teachers' use of motivational messages—making it challenging to draw conclusions based solely on singular observation segments.

LLMs are capable of interpreting human interactions in context‐dependent settings but require access to a comprehensive view of the data. In the studies by Falcon and León (2024) and Metzner et al. (2025), models were trained on one or a few sentences per segment containing either motivational or non‐motivational messages from pre‐ and in‐service teachers. However, these segments excluded preceding and subsequent classroom discourse, limiting the incorporation of contextual information. This approach enabled the authors to label each segment as motivational or non‐motivational but excluded surrounding discourse, potentially omitting contextual cues needed for interpreting more complex messages. For example, detecting linguistic features such as sarcasm can be particularly challenging for LLMs in the absence of contextual information (Chaudhari & Chandankhede, 2017).

One straightforward approach to enhance contextual information in the LLM annotation process is to include teachers' messages that occur immediately before and after each target message. For example, Hou et al. (2024) followed a valuable approach providing the LLM with 16‐min classroom discourse segments for zero‐shot annotation. Although this approach may condense multiple message types into a single rating, it allows the LLM to interpret teachers' messages within a broader classroom context, thereby enriching its annotation decisions.

Moreover, incorporating additional linguistic information, such as audio data capturing tone of voice, could improve LLMs' interpretation of motivational messages. Vrijders et al. (2024) found that the speaker's tone affects the message interpretation—for instance, a controlling tone increased listeners' perceived pressure. In the case of teachers' motivational messages, tone could alter a message's interpretation. For example, a statement like “Today, I have planned an exciting lesson” may seem supportive; however, if delivered in a disinterested tone, it may not be perceived as motivational.

On a similar note, the interplay between teachers' verbal and non‐verbal behaviour can notably impact how classroom discourse is interpreted (Liu, 2021; Young‐Jones et al., 2014). For instance, teachers' autonomy‐supportive messages—like providing justifications for actions and fostering a participative, open learning environment—can be further reinforced through non‐verbal behaviours, such as teachers' active movement in front of the class. Conversely, a controlling learning environment could be established through teachers' autonomy‐controlling messages, such as pressuring statements, as well as non‐verbal behaviours, including distancing from students and reduced physical movement (Young‐Jones et al., 2014). This implies that an enthusiastic teacher statement, for example, may be perceived as less engaging if accompanied by reserved and distant body language.

One approach to integrate teachers' verbal and non‐verbal messages is through multimodal assessment. Hou et al. (2024), for example, developed an ensemble model combining ChatGPT zero‐shot annotations of teachers' encouragement and warmth with a supervised model that incorporated sentiment analysis of teacher transcripts along with video and audio recordings, leveraging facial expression analysis and speech recognition algorithms to assess teachers' emotions. The ensemble model obtained a correlation with human ratings of .51 and suggests promising potential for integrating multiple assessment methods to enhance the detection of verbal and non‐verbal teacher behaviours.

In summary, accurately classifying teachers' motivational messages requires accounting for the complexity of classroom discourse, which may be lost when annotating messages sentence‐by‐sentence. To address this limitation, incorporating contextual information—such as preceding and subsequent utterances, tone of voice, or non‐verbal behaviour—into a multimodal model could enhance interpretation.

### Data biases in training and testing data

Moreover, potential data bias effects that may impact model results (F. Liu et al., 2025), along with broader ethical considerations, represent another key challenge. The data used to train and fine‐tune LLMs may introduce biases, such as historical bias—which reinforces stereotypes related to gender, race, or disability—and representational bias, which can result in the under‐, over‐, or misrepresentation of certain groups, thereby replicating dominant ideologies (Head et al., 2023; Lin et al., 2024). The four studies reviewed used data from Spanish and German schools to test various LLMs, highlighting the potential influence of classroom‐specific factors—such as class composition, teacher and student backgrounds, and lesson context—on model performance. As all studies were conducted in Western, Educated, Industrialised, Rich, and Democratic (WEIRD) countries, this may result in the underrepresentation of diverse educational settings, contributing to representational bias and limiting generalisability. Moreover, LLMs with labelled data can introduce label bias, as human annotators' linguistic and social biases may affect the training process (Head et al., 2023; Hovy & Prabhumoye, 2021). Although all studies reviewed in this work ensured annotator training and calculated inter‐rater reliability, individual sociolinguistic biases—such as misinterpreting sarcasm—or the cultural context the annotators were socialized in could still lead to annotation errors and bias.

Furthermore, using non‐authentic training and testing data can introduce bias and produce unrepresentative outcomes. Alqassab and León (2024), for example, relied on students' open‐ended responses about their teachers' exam‐related motivational messages. However, this approach depends on students' subjective recall rather than on objective transcripts of what the teacher actually said. Consequently, non‐authentic data may not accurately reflect how teachers employ motivational messages in real classrooms. In summary, LLMs may include various types of biases, such as historical, representational, and label biases. Therefore, a critical reflection on the data on which LLMs have been trained, fine‐tuned, and tested is necessary.

### Ethical considerations

Another key challenge involves the ethical considerations associated with using AI‐based methods in educational research, particularly regarding the handling of collected data. Specifically, research involving authentic classroom data must follow data protection regulations to process the sensitive information collected in classroom settings. For instance, classroom transcripts need to be anonymised to protect sensitive data of students and teachers (Meyermann & Porzelt, 2014).

In general, when working with LLMs in educational research, researchers need to be aware of ethical considerations and policy guidelines that ensure the protection of data (European Commission, 2025). If the data are used solely for fine‐tuning an LLM, anonymising the data is recommended, as it ensures that no personal information is included and that no conclusions can be drawn about the participants.

Furthermore, integrating LLMs into educational research raises concerns about environmentally sustainable use. Deploying LLMs requires considerable electricity consumption and produces substantial carbon emissions, partly due to the manufacturing of hardware like GPUs and supercomputers, which involves mining rare earth elements and other metals. Additionally, data collection and management via large data centres, along with the training and fine‐tuning of LLMs, further contribute to their environmental footprint (see Jiang et al., 2024). Therefore, while LLMs offer considerable human‐resource efficiencies in research, their environmental costs must be carefully considered, and strategies to reduce the ecological footprint should guide future development.

In summary, the use of LLMs in educational research presents important ethical challenges, particularly concerning data protection. Moreover, the considerable environmental impact of LLMs highlights the need for sustainable practices and thoughtful implementation to balance research efficiency with ecological responsibility.

### Practical implications

Looking ahead, the use of LLMs to classify teachers' motivational messages is highly promising, particularly as a basis for feedback tools in teacher education and school practice (Reeve & Cheon, 2021; Wang & Chen, 2025). However, several factors must be considered before applying such tools. For instance, technical challenges must be addressed, such as data protection regulations regarding the voices of teachers and students. It is also essential to clearly define the tool's intended purpose and its expected output—ranging from descriptive overviews of teachers' motivational message use to constructive feedback with detailed suggestions on improving teachers' message use, such as increasing supportive and reducing thwarting messages.

In summary, LLM‐based tools offer the potential to provide direct, individualised feedback on teachers' motivational messages—reducing the need for external observers. However, there are several challenges, ranging from developing a reliable model to managing its integration into educational practice.

## CONCLUSION

AI‐based research methods, such as LLMs, have the potential to expand traditional and resource‐intensive teaching behaviour assessment methods. In the scope of this work, we presented four studies which successfully applied LLMs to classify teachers' motivational messages. However, despite these promising results, integrating LLMs into educational research presents several challenges that must be addressed. These include issues related to the quantity and quality of data, the generalisability of LLM‐generated findings, the complexity of classroom interactions, potential biases that may affect outcomes, and ethical considerations surrounding the implementation of LLMs as a research method. For future studies, we encourage integrating LLMs to classify teachers' motivational messages. Yet, critical reflection on the limitations and challenges associated with these methods remains essential. For example, when selecting an open‐source LLM for research purposes or using an existing fine‐tuned LLM to classify teachers' motivational messages, users should be aware that the model's output is based on its initial training and task‐specific fine‐tuning data, which may reflect inherent bias.

## AUTHOR CONTRIBUTIONS

**Olivia Metzner:** Conceptualization; project administration; writing – original draft; writing – review and editing. **Yindong Wang:** Writing – review and editing. **Gerard de Melo:** Writing – review and editing. **Wendy Symes:** Writing – review and editing. **Yizhen Huang:** Writing – review and editing. **Rebecca Lazarides:** Conceptualization; funding acquisition; project administration; resources; supervision; writing – original draft; writing – review and editing.

## CONFLICT OF INTEREST STATEMENT

The authors declare no conflicts of interest.

## Data Availability

Data availability is not applicable to this article as no new data were created or analysed in this comprehensive literature overview.
